# The effect of SARS-CoV-2 infection and vaccination on Th17 and regulatory T cells in a pregnancy cohort in NYC

**DOI:** 10.3389/fimmu.2024.1350288

**Published:** 2024-03-05

**Authors:** Frederieke A. J. Gigase, Mara Graziani, Juliana Castro, Corina Lesseur, Anna-Sophie Rommel, Tammy Flores, M. Mercedes Perez-Rodriguez, Siobhan Dolan, Joanne Stone, Teresa Janevic, Whitney Lieb, Veerle Bergink, Lot D. de Witte

**Affiliations:** ^1^ Department of Psychiatry, Icahn School of Medicine at Mount Sinai, New York, NY, United States; ^2^ Department of Child and Adolescent Psychiatry, Erasmus Medical Center, Rotterdam, Netherlands; ^3^ Department of Human Genetics, Radboud University Medical Center (UMC), Nijmegen, Netherlands; ^4^ Department of Psychiatry, Radboud University Medical Center (UMC), Nijmegen, Netherlands; ^5^ Department of Environmental Medicine and Public Health, Icahn School of Medicine at Mount Sinai, New York, NY, United States; ^6^ Department of Obstetrics and Gynecology, Stamford Health, Stamford, CT, United States; ^7^ Department of Obstetrics, Gynecology and Reproductive Science, Icahn School of Medicine at Mount Sinai, New York, NY, United States; ^8^ Department of Epidemiology, Columbia University Mailman School of Public Health, New York, NY, United States; ^9^ Blavatnik Family Women’s Health Research Institute, Icahn School of Medicine at Mount Sinai, New York, NY, United States; ^10^ Department of Psychiatry, Erasmus Medical Center, Rotterdam, Netherlands

**Keywords:** COVID-19 pandemic, SARS-CoV-2 infection, COVID-19 vaccination, Th17, Treg, ratio

## Abstract

Disturbances in T-cells, specifically the Th17/Treg balance, have been implicated in adverse pregnancy outcomes. We investigated these two T-cell populations following pre-pregnancy and pregnancy SARS-CoV-2 infection and COVID-19 vaccination in 351 participants from a pregnancy cohort in New York City (Generation C; 2020-2022). SARS-CoV-2 infection status was determined via laboratory or medical diagnosis and COVID-19 vaccination status via survey and electronic medical records data. Peripheral blood mononuclear cells (PBMCs) were collected at routine prenatal visits throughout gestation (median 108 days; IQR 67-191 days) with repeated measures for 104 participants (29.6%). T-cell populations CD4+/CD3+, Th17/CD4+, Treg/CD4+ and the Th17/Treg ratio were quantified using flow cytometry. Results showed that inter-individual differences are a main influencing factor in Th17 and Treg variance, however total variance explained remained small (R^2^ = 15-39%). Overall, Th17 and Treg populations were not significantly affected by SARS-CoV-2 infection during pregnancy in adjusted linear mixed models (*p*>0.05), however comparison of repeated measures among SARS-CoV-2 infected participants and non-infected controls suggests a relative increase of the Th17/Treg ratio following infection. In addition, the Th17/Treg ratio was significantly higher after SARS-CoV-2 infection prior to pregnancy (10-138 weeks) compared to controls (β=0.48, *p*=0.003). COVID-19 vaccination was not associated with Th17 and Treg cells. Our findings suggest an impact of SARS-CoV-2 infection on the Th17/Treg ratio, likely depending on severity of infection, yet the observed trends and their potential consequences for pregnancy outcomes require further investigation. Our study contributes to growing evidence that COVID-19 vaccination during pregnancy does not lead to an exacerbated immune response.

## Introduction

Pregnant individuals have increased susceptibility to infections, due to pregnancy-specific maternal immune changes (1, 2). Dysregulation of the maternal immune system has been linked to adverse pregnancy outcomes (3). To understand the impact of the COVID-19 pandemic on pregnant people, it is important to analyze the effect of SARS-CoV-2 infection and COVID-19 vaccination on the maternal immune system. CD4^+^ T cells are part of the adaptive immune response and their activation by antigen-presenting cells leads to expansion and cytokine secretion. CD4^+^ T cells play an important role in establishing the unique immunological state during pregnancy and comprise a range of T-helper (Th) cell populations that are involved in distinct pregnancy processes (4). Specifically, Th17 and regulatory T cells (Treg) are thought to play a crucial role during pregnancy (4–7). Implantation is considered an immune modulatory stimulus, which leads to simultaneous increase of Th17 cells and the activation of Treg cells to dampen an excessive immune response (8). A tightly regulated balance between the Th17 and Treg cells is required for a successful pregnancy while not compromising the maternal immune system’s capacity to fight infection. Treg cells are increased during pregnancy compared to outside of pregnancy (6), and the proportion of Treg cells increases even further later in pregnancy (9). A healthy pregnancy is characterized by a Th17/Treg balance with more Treg compared to Th17 cells (7). Mouse studies have demonstrated the importance of Treg cells during implantation, showing increased fetal rejection following CD25+ Treg cell depletion (10). Also in human studies, disruption in the Th17/Treg ratio, mostly due to decreased presence and function of Treg cells, has been associated with adverse pregnancy outcomes including preterm birth and preeclampsia (7, 11, 12).

SARS-CoV-2 infection has been associated with changes in Th17 and Treg cells in the general population (13–15). Considering the adverse pregnancy outcomes associated with Th17/Treg disruptions, it is crucial to understand T-cell populations in the context of SARS-CoV-2 infection. Yet, few studies have investigated Th17 and Treg cell populations following SARS-CoV-2 infection in the vulnerable pregnant population. One study showed similar percentages of Th17 cells between 17 pregnant participants and 12 non-pregnant controls after SARS-CoV-2 infection (16). A study of 20 pregnant participants with SARS-CoV-2 infection suggested upregulation of T-cell activation pathways compared to pregnant controls (17). The largest pregnancy study to date reported a lower percentage of Treg cells and a decreased Treg/Th17 ratio in 56 pregnant participants after SARS-CoV-2 infection in the first and third trimester compared to 61 healthy pregnant controls (18). The authors found no difference in the Th17 cell population between SARS-CoV-2 infected and healthy pregnant participants (18). A third study in eight pregnant people showed that SARS-CoV-2 infection resulted in virus-specific inducible Treg cells (19). Existing studies on SARS-CoV-2 infection during pregnancy and the effect on Th17 and Treg cells are limited by small sample sizes (range n=8-117), varying control groups of both pregnant and non-pregnant controls, and their disregard of the effect of gestational age, infection severity, pandemic timing and time between infection and vaccination in univariate comparisons. Whilst several studies have looked into the safety of COVID-19 vaccination during pregnancy, little is known about the effect of COVID-19 vaccination on Th17 and Treg populations.

Here, we investigated the association between prenatal SARS-CoV-2 infection and COVID-19 vaccination and the percentages of Treg and Th17, its plasma component IL-17A and the Th17/Treg ratio in the Generation C study, a multi-ethnic pregnancy cohort conducted at the Icahn School of Medicine at Mount Sinai in New York City (20). This is the largest cohort to date, including 351 pregnant participants, with unbiased sampling throughout gestation and repeated measures to assess the influence of SARS-CoV-2 infection and COVID-19 vaccination on T-cell populations over time.

## Methods

### Study design

This study is embedded in the Generation C study (n=3,157), a multi-ethnic pregnancy cohort in New York City conducted between April 2020 and February 2022 (20). The current study is part of a modified study protocol, Generation C II, which includes the collection of peripheral blood monocyte cells (PBMCs) and was initiated on April 19, 2021. Of 541 participants included in Generation C II, PBMC samples were successfully collected in 398 individuals (73.6%) between 19 April 2021 and 15 September 2022. In the remaining 143 individuals, PBMC sample collection was unsuccessful due to low or no blood volume at blood draw, blood clotting (e.g. if CPT tubes were not inverted), methodological issues including the availability of a centrifuge for 1 hour (brake-off), or extensive time between sample collection and processing (e.g. sample left overnight, or on holidays), in which case the sample was discarded. These participants were excluded (n=143). In addition, participants were excluded in case of multiple pregnancy (n=9), if the collected sample had incomplete T-cell outcome data (n=43) or if the %Th17 and/or %Treg was an outlier (n=4). Included participants were compared to excluded populations in **Supplementary Table 1**. A flowchart is shown in **Supplementary Figure 1**.

### SARS-CoV-2 infection and COVID-19 vaccination status

SARS-CoV-2 infection status was determined based on 1) anti-Spike and anti-Nucleocapsid antibody presence measured in all participants, 2) a positive RT-PCR, or 3) diagnosis by a medical provider reported in the electronic medical records (EMR). The date of specimen collection, positive RT-PCR test, or date of EMR report was considered the date of SARS-CoV-2 infection and used to determine timing between SARS-CoV-2 infection and sampling. Pregnant people were considered never infected if they had no indication of SARS-CoV-2 infection according to these determinants. Participants were divided into three groups: 1) evidence of SARS-CoV-2 infection at any point during gestation, 2) evidence of SARS-CoV-2 infection prior to gestation and 3) no evidence of prior SARS-CoV-2 infection. COVID-19 vaccination status was ascertained through 1) a survey which enquired about the participant’s vaccination status, vaccination time point(s), and brand of vaccination; and 2) data extracted from the New York City Immunization Registry (NYCIR) through patients’ EMR, as described previously (21). Participants were divided into three groups: 1) vaccinated during pregnancy if they had received their first dose of the Janssen (Ad26.COV2.S), Moderna (mRNA-1273) or Pfizer-BioNTech (BNT162b2) vaccine at any point during pregnancy, 2) vaccinated prior to pregnancy if they received the first dose of the vaccine prior to gestation, and 3) never vaccinated if they had no evidence of receiving a vaccine, or if the PBMC sample was obtained prior to vaccination. The date of vaccination was used to determine timing between COVID-19 vaccination and sampling.

### Blood collection and processing

As part of routine prenatal visits, blood was collected in one 4ml sodium heparin CPT tube (BD Vacutainer^®^ CPT™) by peripheral venipuncture. PBMC collection was performed only if serum collection had been completed, i.e., CPT tubes were collected only if EDTA tubes had already been collected. In case of low blood draw volume, only EDTA tubes were collected but no PBMC collection was done. Tubes were centrifuged at 1,800g for 20 minutes at room temperature with the brake-off. After plasma collection, the buffy coat containing PBMCs was collected and washed two times in phosphate-buffered saline (PBS; pH 7.4, Gibco™) at 300g for 15 and 10 minutes respectively at room temperature. Plasma was collected and stored at -80°C until use. Viable cells were counted with Trypan blue (0.4%, Gibco™). PBMC were resuspended in CryoStor^®^ CS10 and stored in Nalgene Mr. Frosty container overnight at -80°C and then transferred to liquid nitrogen. IL-17A was measured in plasma samples using the High Sensitivity T-cell Discovery Array 3-Plex (Millipore, St. Charles, MO, USA) at Eve Technologies using the Bio-Plex™ 200 system (Bio-Rad Laboratories, Inc., Hercules, CA, USA). For participants included in the current study, a minimum of one and a maximum of two samples were collected.

### Flow cytometry

Cryopreserved PBMC samples were thawed, resuspended in 100uL of FACS buffer (4% EDTA, 5% BSA in PBS pH 7.4) and washed at 1,400 rpm for 2 minutes at room temperature. Live/dead staining was performed using Zombie Violet™ Fixable Viability Kit (Biolegend, San Diego, CA) in the dark for 15 minutes at room temperature. Extracellular staining was performed using the human antibodies BV650 anti-CD3 (BD Bioscience, San Diego, CA), Vio-Bright-R720 anti-CD4 (Miltenyi), PE anti-CD25 (Biolegend, San Diego, CA), APC-Cy7 anti-CD127 (Biolegend, San Diego, CA), Vio-Bright-FITC anti-CXCR3 (Miltenyi), APC anti-CCR10 (Miltenyi), PE-Cy7 anti-CCR4 (Biolegend, San Diego, CA), PE-Vio-615 anti-CCR6 (Miltenyi) in the dark for 30 minutes at 4°C. The cells were washed in the FACS buffer at 1600 rpm for 2 minutes at room temperature. Cells were resuspended in the FACS buffer and analyzed using the Attune NxT Flow Cytometer (median recorded events: 194,956, interquartile range: 174,576). A compensation procedure was performed using compensation beads (Invitrogen™UltraComp eBeads™). Th17 and Treg cell populations were identified according to an adaptation of existing protocols described by Zhong et. Al. and Miltenyi (22). The gating strategy was as follows: 1) CD4^+^ T-cells were gated using Side Scatter and CD3^+^; 2) Th17 cells were identified using CD4^+^, CCR6^+^, CCR4^+^, CXCR3^-^, CCR10^-^ (22) and 3) Treg were identified using CD4^+^, CD25^+^, and CD127^-^ (Miltenyi) (**Supplementary Figure 2**). Fluorescence Minus One (FMO) controls were performed for each antibody and was used to set the gates for CCR6, CCR4, CD127, CCR10 and CXCR3. Data were analyzed by FlowJo software for Mac (23). T-cell outcomes included in the current study are the T-cell population percentages, including CD4^+^/CD3^+^, Th17/CD4^+^, Treg/CD4^+^ and the Th17/Treg ratio.

### Statistical analysis

Analyses were performed using the R Statistical Software (version 4.1.2) (24). Visualizations were created with the ggplot2 package (25). Demographics and outcomes were compared between groups using the Mann-Whitney U test and Kruskal-Wallis test for continuous variables and Chi-square test for categorical variables for non-normally distributed variables (Shapiro-Wilk *p*<0.001). Principal component analysis (PCA) was performed to identify outlier samples (>3 standard deviations from the grand mean of PC1) and to visualize factors that showed a high contribution to the immunological T cell profile. Variance partition analysis was performed to asses to which factors the variation in percentage of CD4^+^, Th17 and Treg cells and the Th17/Treg ratio can be attributed, using the ‘variancePartition’ package in R (26). Linear mixed modeling was performed to investigate the association between 1) SARS-CoV-2 infection prior to and during pregnancy (reference group = no evidence of SARS-CoV-2 infection) and 2) COVID-19 vaccination prior to and during pregnancy (reference group = no evidence of COVID -19 vaccination) and outcomes 1) percentage of CD4^+^ cells in CD3^+^ cells, 2) percentage of Th17 cells in CD4^+^ cells, 3) percentage of Treg cells in CD4^+^ cells, 4) the ratio between number of Th17 and Treg cells (Th17/Treg) and 5) plasma IL-17A levels (pg/mL). Participant was modeled as a random effect to control for repeated measures. The covariates maternal age (<35, ≥35); BMI (kg/m^2^: underweight (<18), normal weight (18–25), overweight (25–30), obese (>30)); race/ethnicity (Black, Asian, Hispanic, White, Other); parity (nulliparous/multiparous); insurance (private/self-pay, public); pre-pregnancy diabetes (yes/no); pre-pregnancy hypertension (yes/no); fetal sex (male/female); time since the start of the pandemic (weeks between first day of pregnancy and date of first SARS-Cov-2 infection in NYC on March 1, 2020) and the technical covariates gestational age at sampling (days); experiment batch and compensation batch were included as fixed effects. Additionally, in SARS-CoV-2 infection models, COVID-19 vaccination status and time (days) between the first evidence of infection and sampling were included as fixed effects. In COVID-19 vaccination models, SARS-CoV-2 infection status and time (days) between the first evidence of vaccination and sampling were included as fixed effects. The marginal variance (i.e., variance explained by fixed effects), and the conditional variance (taking into account the individual, modeled as a random intercept) are reported for the linear mixed models. The R packages lme4 and lmertest were used to fit the models (27). Collinearity was assessed using the variance inflation factor (VIF), which was below 2 for all covariates, thus indicating no collinearity problems (28). We further investigated the impact of SARS-CoV-2 infection on Th17 and Treg populations in a repeated measures analysis. Participants with a repeated measurement prior to and after SARS-CoV-2 infection were selected (n=22) and T-cell outcomes were compared prior to and after SARS-CoV-2 infection using a repeated measures ANOVA, controlled for covariates listed above. Trajectories were compared to a control group of gestational age matched non-infected controls (ratio 1:4, n=88). The repeated measures analysis was not performed among COVID-19 vaccinated participants due to the low number of vaccinated participants with repeated measures during pregnancy (n=4). Lastly, to investigate the effect of time between SARS-CoV-2 infection and COVID-19 vaccination and sampling, participants were grouped according to the time between first evidence of SARS-CoV-2 infection and first sample collection, as well as time between first evidence of COVID-19 vaccination and first sample collection, as follows: 0-3 months, 3-6 months, 6-9 months, 9-12 months, >12 months. In linear regression analyses, each group was compared to the reference group of never infected/vaccinated participants, while controlling for covariates listed above. Analyses were corrected for multiple testing using the Benjamini-Hochberg method (29). Corrected p-values are shown.

## Results

### Characteristics of the sample

**Figure 1:**
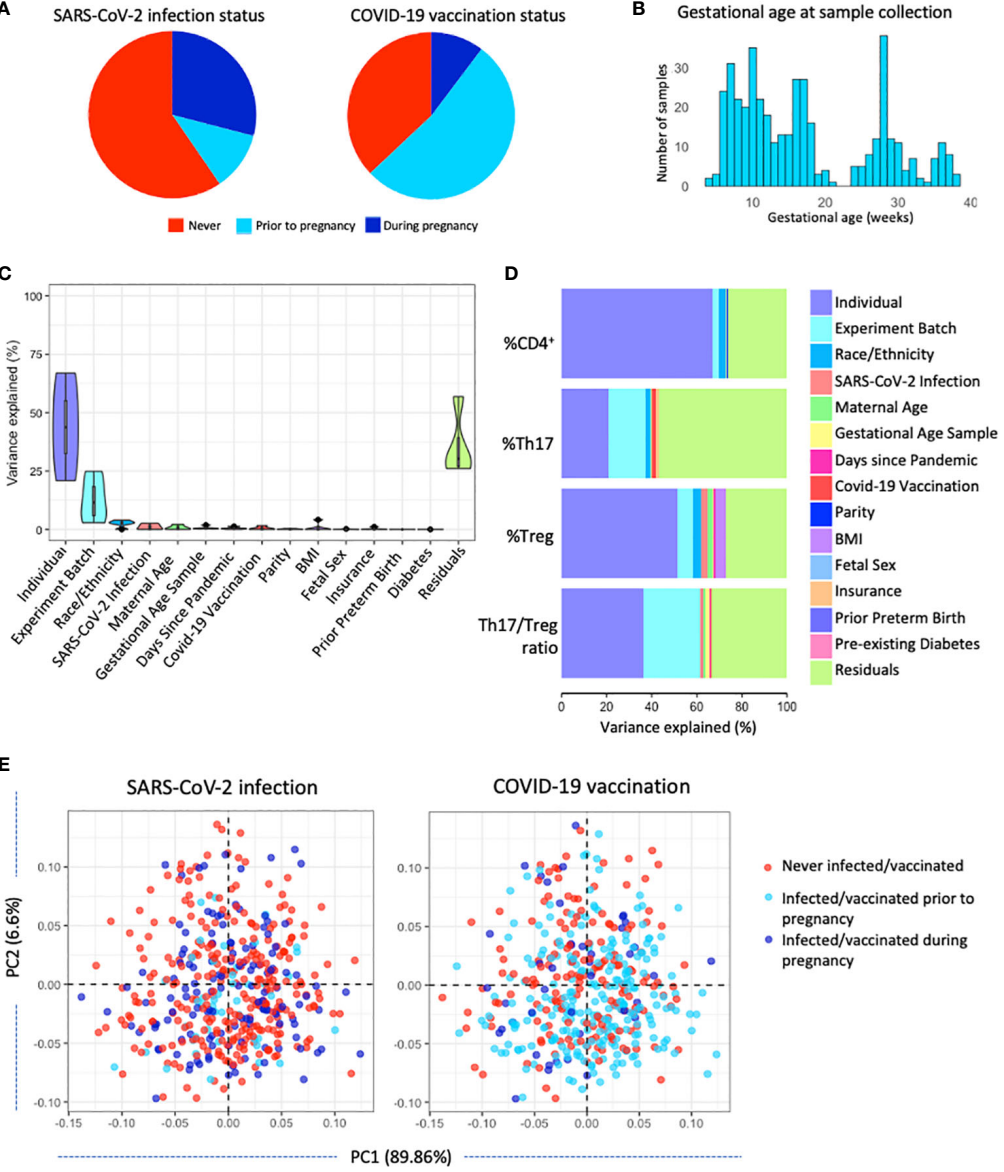
Characterizing T-cell populations in a large pregnancy cohort in NYC (n=351). **(A)** SARS-CoV-2 infection status and COVID-19 vaccination status was determined for all participants. **(B)** Gestational age at sampling: samples were collected throughout gestation at a median of 108 days (IQR 67-191 days). **(C)** Variance partition analysis of the variance in T cell populations explained by various maternal, technical, and COVID-19 pandemic-related factors. **(D)** Variance partition results showing the variance explained by various maternal, technical, and COVID-19 pandemic-related factors in each T-cell population. **(E)** Principal component analysis showing the distribution of SARS-CoV-2 infection status and COVID-19 vaccination status across T-cell populations.

Of 351 included participants, 102 (29.1%) were infected with SARS-CoV-2 during pregnancy, with equal distribution of infected cases across trimesters (**Table 1**, **Figure 1A**). Of participants infected during pregnancy one participant (1%) was hospitalized due to SARS-CoV-2 infection. In total 36 (10.3%) participants received the first dose of a COVID-19 vaccine during pregnancy while 185 (52.7%) received their first dose of a COVID-19 vaccine prior to pregnancy, with significantly more people having received a dose of a COVID-19 vaccine during pregnancy among the infected group (*p*<0.001). The majority of participants were below 35 years old (n=211, 60.1%) and had normal pre-pregnancy BMI (n=153, 43.6%). Race/ethnicity did not differ significantly between infection groups (**Table 1**). Included participants did not differ from excluded participants in terms of demographics, yet SARS-CoV-2 infection and COVID-19 vaccination was more prevalent (*p*<0.01; **Supplementary Table 1**). Samples were collected throughout gestation at a median of 108 days (IQR 67-191 days) (**Figure 1B**). Of 351 participants, a second sample was collected for 104 participants (29.6%) and a total of 455 samples were included in the current analysis (**Supplementary Figure 3**).

**Table 1:** Characteristics of 351 participants of the Generation C pregnancy cohort.

Characteristic	All participants (n=351)	Never infected (n=209)	SARS-CoV-2 infection prior to pregnancy (n=40)	SARS-CoV-2 infection during pregnancy (n=102)	*P*-value*
Maternal Age, median (IQR)					**0.022**
• **<35** • **35 and up** • **Unknown**	211 (60.1) 97 (27.6) 43 (12.3)	114 (54.5) 60 (28.7) 35 (16.7)	27 (67.5) 11 (27.5 2 (5.0)	70 (68.6) 26 (25.5) 6 (5.9)	
Race/ethnicity, n (%)					0.087
• **Asian** • **Black** • **Hispanic** • **White** • **Other** • **Unknown**	29 (8.3) 51 (14.5) 84 (23.9) 92 (26.2) 15 (4.3) 80 (22.8)	18 (8.6) 34 (16.3) 54 (25.8) 57 (27.3) 5 (2.4) 41 (19.6)	2 (5.0) 9 (22.5) 9 (22.5) 8 (20.0) 4 (10.0) 8 (20.0)	9 (8.8) 8 (7.8) 21 (20.6) 27 (26.5) 6 (5.9) 31 (30.4)	
Parity, n (%)					0.123
• **Nulliparous** • **Multiparous**	144 (41) 207 (59.0)	90 (43.1) 119 (56.9)	20 (50.0) 20 (50.0)	34 (33.3) 68 (66.7)	
Pre-pregnancy BMI, n (%)					0.192
• **Underweight (<18)** • **Normal weight (18-24.9)** • **Overweight (25-30)** • **Obese (>30)** • **Unknown**	11 (3.1) 153 (43.6) 78 (22.2) 100 (28.5) 9 (2.6)	6 (2.9) 100 (47.8) 46 (22.0) 51 (24.4) 6 (2.9)	2 (5.0) 19 (47.5) 4 (10.0) 14 (35.0) 1 (2.5)	3 (2.9) 34 (33.3) 28 (27.5) 35 (34.3) 2 (2.0)	
Insurance, n (%)					0.963
• **Private/Self-pay** • **Public**	248 (70.7) 103 (29.3)	147 (70.3) 62 (29.7)	29 (72.5) 11 (27.5)	72 (70.6) 30 (29.4)	
Pre-pregnancy diabetes, n (%)	4 (1.1)	4 (1.9)	0 (0.0)	0 (0.0)	0.253
Pre-pregnancy hypertension, n (%)	19 (5.4)	10 (4.8)	2 (5.0)	7 (6.9)	0.743
Gestational diabetes, n (%)	41 (11.7)	23 (11.0)	4 (10.0)	24 (23.5)	0.825
Gestational hypertension, n (%)	49 (14.0)	26 (12.4)	3 (7.5)	20 (19.6)	0.152
Preterm birth, n (%)	32 (9.1)	18 (8.6)	3 (7.5)	11 (10.8)	0.766
Female fetus, n (%)	151 (43.0)	85 (40.7)	20 (50.0)	46 (45.1)	0.886
Trimester of infection, n (%)					NA
• **1** • **2** • **3**				37 (36.3) 31 (30.4) 34 (33.3)	
Hospitalized for COVID-19 illness			0 (0.0)	1 (1.0)	0.486
COVID-19 vaccination, n (%)					**<0.001**
• **No proof of vaccination prior to** ** sampling** • **First dose prior to pregnancy** • **First dose during pregnancy prior** ** to sampling**	130 (37.0) 185 (52.7) 36 (10.3)	89 (42.6) 111 (53.1) 9 (4.3)	12 (30.0) 24 (60.0) 4 (10.0)	29 (28.4) 50 (49.0) 23 (22.5)	

Characteristics are compared between participants with no evidence of prior SARS-CoV-2 infection (n=209) and participants with SARS-CoV-2 infection prior to (n=40) and during pregnancy (n=102).

*P-value indicates statistical comparison between infection groups using Chi-square test for categorical variables. Bold p-values indicate statistical significance at the alpha <0.05 level. NA, Not applicable.

### Drivers of T-cell populations

To better understand what factors influence Th17 and Treg cells we performed a variance partition analysis. Most variance in the T-cell populations was attributed to residual factors (25-50%) other than the maternal, technical, and pandemic-related factors investigated in this analysis, including SARS-CoV-2 infection and COVID-19 vaccination. In addition, by having repeated measures, we identified that a substantial proportion of the variance in T-cell populations was explained by the individual, mostly in total CD4^+^ and Treg cell populations (20-65%). Part of the variance in T-cell populations can be attributed to technical factor *experiment batch*, as well as demographic factors *maternal age* and *race/ethnicity* and pandemic-related factors *SARS-CoV-2 infection status*, *days since the start of the pandemic* and *COVID-19 vaccination status* (**Figure 1C**). The variance explained by maternal and technical factors on each individual outcome points to *race/ethnicity*, *insurance status*, and *COVID-19 vaccination status* as potential drivers of the percentage of Th17 in the CD4^+^ population, and implicates *race/ethnicity*, *maternal age*, *BMI*, and *SARS-CoV-2 infection status* as potential drivers of the percentage of Treg cells in the CD4^+^ population (**Figure 1D**). However, variance explained by these factors remains minor as confirmed by multivariable models (R^2^ = 15-39%; **Table 2**). SARS-CoV-2 infection status and COVID-19 vaccination status did not show clear clustering patterns across the first and second principal component, suggesting no clear association with T-cell populations (**Figure 1E**).

**Table 2:** Linear mixed models were performed to assess the association between SARS-CoV-2 infection and COVID-19 vaccination with T-cell populations.

	%CD4^+^		%Th17		%Treg		Th17/Treg ratio		IL-17A (pg/mL)	
	β	*P*-value	β	*P*-value	β	*P*-value	β	*P*-value	β	*P*-value
SARS-CoV-2 infection analyses										
Unadjusted models										
Infected during pregnancy^a^	0.27 (-1.84; 2.37)	0.804	-0.02 (-0.48; 0.44)	0.934	-0.19 (-0.70; 0.32)	0.465	0.02 (-0.08; 0.11)	0.729	-0.50 (-3.16; 2.17)	0.714
Infected prior to pregnancy^a^	-0.21 (-3.28; 2.85)	0.892	0.24 (-0.45; 0.93)	0.499	-1.00 (-1.75; -0.25)	**0.009**	0.21 (0.07; 0.35)	**0.004**	2.71 (-1.19; 6.60)	0.172
Adjusted models^b^										
Infected during pregnancy^a^	0.94 (-2.01; 3.90)	0.531	0.31 (-0.36; 0.98)	0.361	-0.23 (-0.94; 0.49)	0.529	0.10 (-0.03; 0.23)	0.124	-2.69 (-6.59; 1.21)	0.176
Infected prior to pregnancy^a^	-2.4 (-9.68; 4.88)	0.517	1.33 (-0.34; 2.99)	0.118	-1.32 (-3.10; 0.45)	0.144	0.48 (0.16; 0.80)	**0.003**	-8.48 (-18.48; 1.52)	0.096
Marginal R^2^/Conditional R^2^ (%)	18/80		28/54		27/70		39/60		25/75	
COVID-19 Vaccination analyses										
Unadjusted models										
Vaccinated during pregnancy^c^	-1.38 (-4.71; 1.94)	0.414	-0.33 (-1.06; 0.40)	0.369	0.40 (-0.41; 1.22)	0.334	-0.11 (-0.27; 0.05)	0.174	0.14 (-4.12; 4.41)	0.947
Vaccinated prior to pregnancy^c^	1.42 (-0.55; 3.40)	0.158	-0.85 (-1.28; -0.42	**<0.001**	-0.59 (-1.07; -0.10)	**0.018**	-0.10 (-0.19; -0.01)	**0.033**	0.34 (-2.19; 2.86)	0.793
Adjusted models^d^										
Vaccinated during pregnancy^c^	-2.02 (-6.99; 2.94)	0.424	-0.28 (-1.40; 0.85)	0.629	-0.30 (-1.51; 0.91)	0.627	-0.07 (-0.29; 0.15)	0.535	0.30 (-6.58; 7.17)	0.932
Vaccinated prior to pregnancy^c^	-1.43 (-7.74; 4.87)	0.655	-0.98 (-2.52; 0.56)	0.213	-0.11 (-1.73; 1.51)	0.895	-0.29 (-0.59; -0.01)	0.06	0.67 (-8.23; 9.58)	0.882
Marginal R^2^/Conditional R^2^ (%)	17/78		27/57		25/67		37/63		15/73	

^a^Reference group = no evidence of prior SARS-CoV-2 infection ^b^SARS-CoV-2 infection models were adjusted for maternal age (<35, >35), BMI [underweight (<18), normal weight (18–25), overweight (25-30), obese (>30)], race/ethnicity (Black, Asian, Hispanic, White, Other), parity (nulliparous/multiparous), insurance (private/self-pay, public), pre-pregnancy diabetes (yes/no), pre-pregnancy hypertension (yes/no), fetal sex (male/female), time since the start of the pandemic (weeks since first day of pregnancy and March 1, 2020), time between first evidence of SARS-CoV-2 infection and sampling, COVID-19 vaccination status (no evidence of prior vaccination, first dose prior to pregnancy, first dose during pregnancy) and technical covariates gestational age at sampling (days), experiment batch and compensation batch. ^c^Reference group = no evidence of prior COVID-19 vaccination ^d^COVID-19 vaccination models were adjusted for maternal age (<35, >35), BMI [underweight (<18), normal weight (18-25), overweight (25-30), obese (>30)], race/ethnicity (Black, Asian, Hispanic, White, Other), parity (nulliparous/multiparous), insurance (private/self-pay, public), pre-pregnancy diabetes (yes/no), pre-pregnancy hypertension (yes/no), fetal sex (male/female), time since the start of the pandemic (weeks since first day of pregnancy and March 1, 2020), time between first evidence of COVID-19 vaccination and sampling, SARS-CoV-2 infection status (no evidence of prior infection, infected prior to pregnancy, infected during pregnancy) and technical covariates gestational age at sampling (days), experiment batch and compensation batch. Bold p-values indicate significant p-values.

### Comparison of infection and vaccination groups

**Figure 2:**
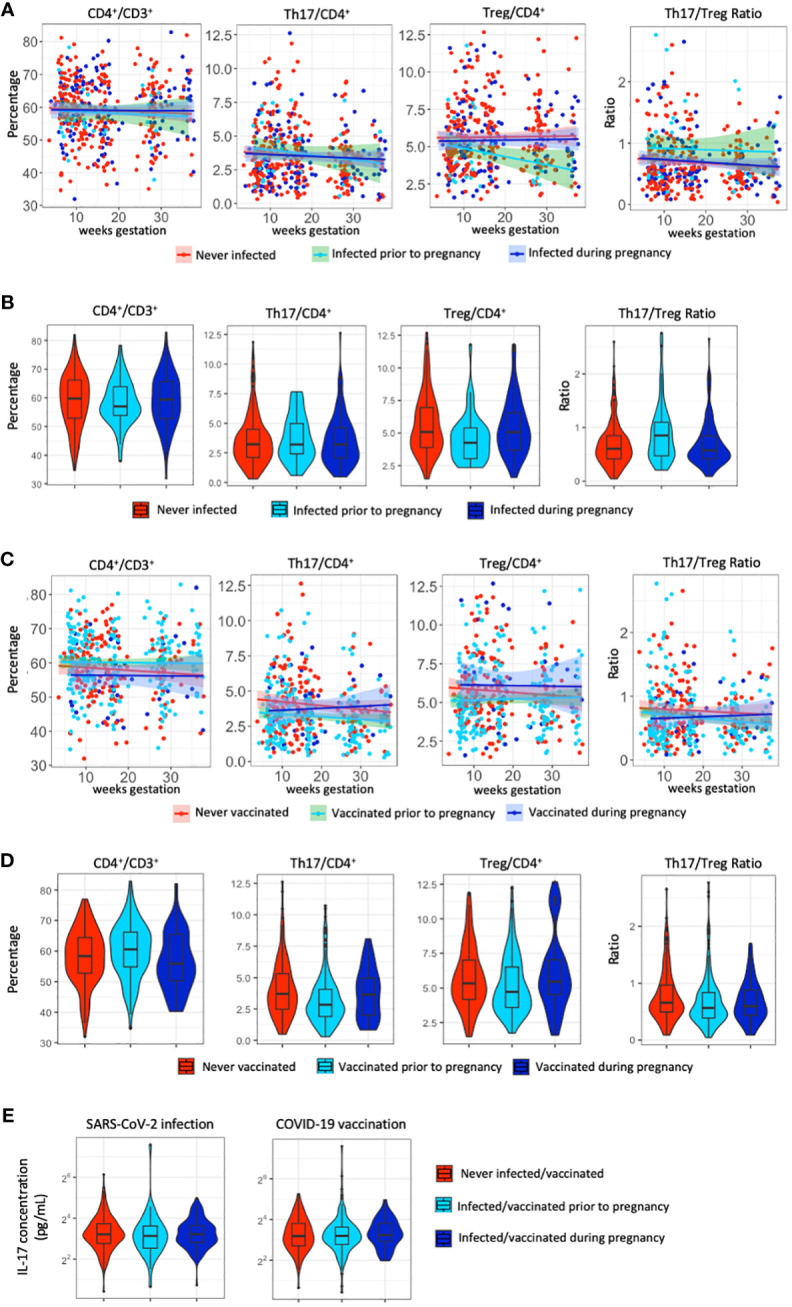
T-cell populations and IL-17A following SARS-CoV-2 infection and COVID-19 vaccination. **(A)** Distribution of the percentage of CD4^+^, Th17, Treg cells and the Th17/Treg ratio throughout gestation across SARS-CoV-2 infection status. **(B)** Violin plots of the distribution of the percentage of CD4^+^, Th17, Treg cells and the Th17/Treg ratio across SARS-CoV-2 infection status. **(C)** Distribution of the percentage of CD4^+^, Th17, Treg cells and the Th17/Treg ratio throughout gestation across COVID-19 vaccination status. **(D)** Violin plots of the distribution of the percentage of CD4^+^, Th17, Treg cells and the Th17/Treg ratio across COVID-19 vaccination status. **(E)** Violin plots of the distribution of IL-17A levels (pg/ml) per SARS-CoV-2 infection status and COVID-19 vaccination status.

Distribution of T-cell populations across gestational age show similar patterns across SARS-CoV-2 infection status (**Figure 2A**). Percentages of T cell populations are not significantly different between participants with no evidence of prior SARS-CoV-2 infection and those with SARS-CoV-2 infection prior to or during pregnancy (*p*>0.05), except for the percentage of Treg cells which is significantly lower (*p*=0.045) among participants infected prior to pregnancy (**Figure 2B**; **Supplementary Table 2**). T-cell populations across gestational age show varying patterns across COVID-19 vaccination status (**Figure 2C**) and are significantly different between participants who have no proof of receiving a COVID-19 vaccination and those who received the first dose of any COVID-19 vaccine prior to or during pregnancy (*p*<0.05) (**Figure 2D**; **Supplementary Table 3**). The percentage of CD4^+^, Th17, Treg cells and the Th17/Treg ratio is significantly lower after vaccination prior to pregnancy compared to those with no proof of vaccination (**Supplementary Table 3**). IL-17A, the plasma component of Th17 cells, was not significantly different between SARS-CoV-2 infected and non-infected pregnant participants (**Supplementary Table 2**), nor between COVID-19 vaccinated and non-vaccinated pregnant participants (**Supplementary Table 3**), as visualized in **Figure 2E**.

### Multivariable analyses

After controlling for covariates, we find no significant association between SARS-CoV-2 infection during pregnancy and percentages of CD4^+^, Th17, Treg cells, IL-17A levels nor in the Th17/Treg ratio (**Table 2**). SARS-CoV-2 infection prior to pregnancy is associated with an increased Th17/Treg ratio (β=0.48, *p*=0.003) compared to non-infected participants. We hypothesized that the association between SARS-CoV-2 infection prior to pregnancy and the Th17/Treg ratio might be dependent on the time between pre-pregnancy SARS-CoV-2 infection and sampling during pregnancy. In multivariable analyses, we found no association of time between infection or vaccination and sampling on the Th17/Treg ratio. We further investigated the effect of time between SARS-CoV-2 infection and sampling by comparing infection timing groups (0-3 months, 3-6 months, 6-9 months, 9-12 months, > 12 months between SARS-CoV-2 infection and sampling) to a reference group of non-infected participants. The Th17/Treg ratio, but not percentages of CD4^+^, Th17, Treg cells, was significantly higher among participants with SARS-CoV-2 infection more than 12 months prior to sampling (*p*=0.020) (**Supplementary Figure 4A**, **Supplementary Table 4A**). COVID-19 vaccination prior to or during pregnancy was not significantly associated with T cell populations nor IL-17A levels in adjusted analyses (**Table 2**). Percentages of CD4^+^, Th17, Treg cells and the Th17/Treg ratio were not significantly different between vaccine timing groups (0-3 months, 3-6 months, 6-9 months, 9-12 months, > 12 months between COVID-19 vaccination and sampling) and a reference group of never vaccinated participants (**Supplementary Figure 4B**; **Supplementary Table 4B**).

### Repeated measures analyses

**Figure 3:**
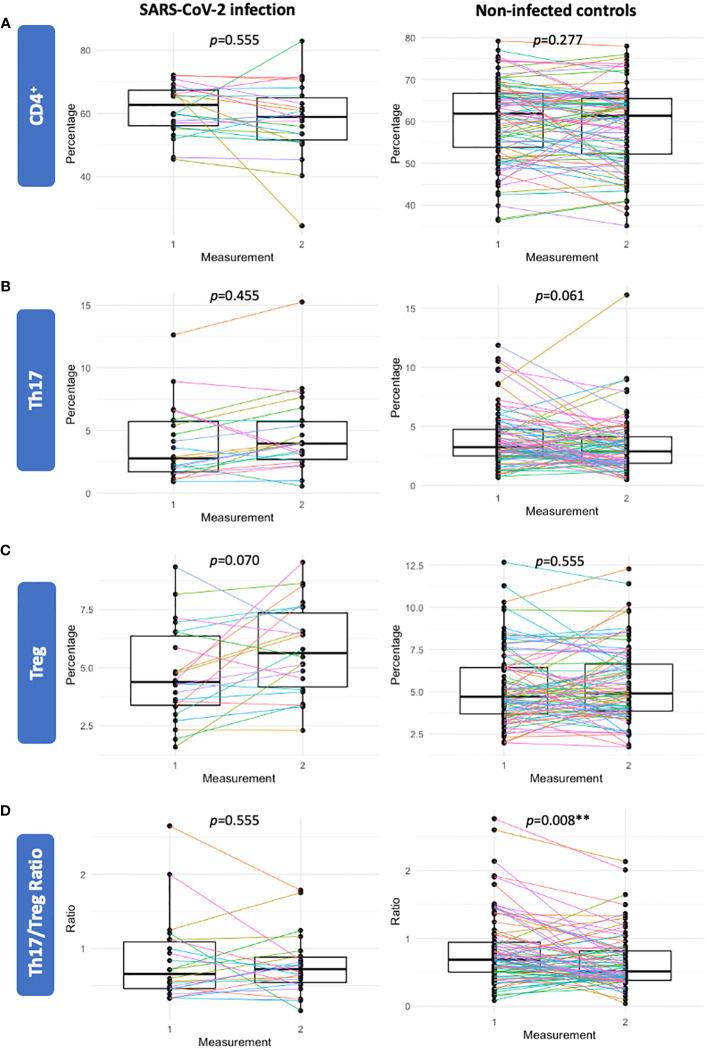
Sensitivity analysis of participants with a repeated measure prior to and after SARS-CoV-2 infection (n=22) (left panels) and a gestational age matched control group of non-infected participants (n=88) (right panels). Percentages of CD4+ **(A)**, Th17 **(B)**, Treg **(C)** and the Th17/Treg ratio **(D)** were not significantly different following SARS-CoV-2 infection. In the gestational age matched control group of non-infected participants (n=88), the Th17/Treg ratio was significantly lower in the second sample compared to the first measurement. Corrected p-values are shown (Benjamini Hochberg).

As inter-individual factors were found to be an important driver of Th17 and Treg variance, we further investigated the trajectories of CD4+, Th17, Treg cells and the Th17/Treg ratio in a subset of participants with repeated measurements. Among gestational age matched non-infected controls (n=88), the Th17/Treg ratio was significantly lower at the second measurement (*p*=0.008), which was largely driven by a decrease in Th17 cells (**Figures 3B, D**). Among participants with a repeated measure prior to and after SARS-CoV-2 infection (n=22), this trend was not observed and percentages of CD4^+^, Th17, Treg cells and the Th17/Treg ratio were not significantly different after SARS-CoV-2 infection compared to prior to infection (**Figures 3A–D**).

## Discussion

Here, we investigated the effect of SARS-CoV-2 infection and COVID-19 vaccination on various T-cell populations and IL-17A levels in maternal blood samples collected throughout gestation in a large, diverse pregnancy cohort in NYC. Results showed that overall, the percentage of CD4^+^, Th17 and Treg cell populations, the Th17/Treg balance and IL-17A levels were not significantly affected by SARS-CoV-2 infection during pregnancy. As inter-individual factors were found to be an important driver of Th17 and Treg cells, we further investigated the impact of SARS-CoV-2 infection on T-cell trajectories among participants with a repeated measurement and observed an increasing trend of the Th17/Treg ratio following SARS-CoV-2 infection compared to non-infected controls. Moreover, the Th17/Treg ratio was significantly higher following SARS-CoV-2 infection prior to pregnancy compared to non-infected participants, in particular among participants infected more than 12 months prior to sampling. COVID-19 vaccination was not associated with Th17 and Treg changes compared to non-vaccinated pregnant controls.

Few studies have investigated the effects of SARS-CoV-2 infection and COVID-19 vaccination on Th17 and Treg populations in relation to pregnancy. Existing results are ambiguous and have reported no difference in Th17 cells after SARS-CoV-2 infection compared to outside of pregnancy (16), nor when compared to healthy pregnant participants (18), on the one hand, and on the other hand have found decreased Treg and Treg/Th17 ratio (thus increased Th17/Treg ratio) after first and third trimester SARS-CoV-2 infection (18). Our finding of no change in Th17 cells is in line with the largest study to date (n=117) (18). In addition, we observed no changes in the percentage of Treg and the Th17/Treg ratio in response to SARS-CoV-2 infection during pregnancy in the overall sample. However, comparison of repeated measures among SARS-CoV-2 infected participants and non-infected controls suggests an increase of the Th17/Treg ratio following infection, which is in line with their finding (18). Our finding that the percentage of Th17 cells and the Th17/Treg ratio decreased over time in normal gestation, but this trend was not observed after SARS-CoV-2 infection, suggests that SARS-CoV-2 infection may attenuate the decrease in Th17 and the Th17/Treg ratio. It should be noted that the subset of participants with repeated measures prior to and after the event was small (n=22) and further validation in longitudinal studies is warranted, especially considering our finding of inter-individual variation in Th17 and Treg populations. Several factors should be considered when comparing existing findings. A control group of non-pregnant SARS-CoV-2 infected participants was used in a prior study (16), which may complicate the disentanglement of the effects of SARS-CoV-2 infection from pregnancy specific immunological changes occurring throughout gestation. Additionally, even though the virus-specific T-cell responses to SARS-CoV-2 in T-cells isolated from PBMCs of SARS-CoV-2 infected participants was found to be ongoing months after infection (19), it is likely that the effect on Treg and Th17 cell percentages is transient. As time between SARS-CoV-2 infection or COVID-19 vaccination and sampling may influence the extent to which T-cell populations are affected, we controlled for the time between SARS-CoV-2 infection and COVID-19 vaccination and sampling. Lastly, differences in T cell characterization may affect findings. Kulhan et al. (2023) identified Treg cells by CD3^+^CD4^+^CD25^+^FOXP3^+^ expression. For Th17 detection, Kulhan et al. (2023) evaluated CD3^+^CD4^+^CCR6^+^CCR4^+^CXCR3^-^ cells, whereas Tartaglia et al. (2022) assessed CD4^+^CCR4^+^CCR6^+^ cells. Of note, the detection of Th17 and Treg populations through cell surface marker expression is one of multiple detection methods and it is at present unclear how different methods may affect the results.

Findings outside of pregnancy suggest that the extent to which T-cell populations are affected by SARS-CoV-2 infection is associated with disease severity. A higher percentage of Th17 cells at baseline was reported in patients who progress to critical COVID-19 (14). Furthermore, Treg cells showed increasing proliferation from mild to severe SARS-CoV-2 infected patients (15). Increased proliferation, activation and cytotoxicity of T-cells was reported in severe SARS-CoV-2 infected patients (13). Previous studies in pregnancy have included pregnant participants reporting at least mild clinical symptoms (16), possibly indicating slightly more severe illness compared to the current study, or did not report infection characterization (18). The current study applied unbiased sampling and we captured both symptomatic and asymptomatic cases. Here, T-cell populations were assessed in the absence of severe disease, as only one participant (1%) was hospitalized following SARS-CoV-2 infection. Interestingly, participants infected prior to pregnancy, particularly more than 12 months prior to sampling, had a significantly higher Th17/Treg ratio at the time of sampling. Although this group is small, we speculate that this may be due to the more severe SARS-CoV-2 virus variant early on in the pandemic, around the time when these participants were infected. However, further validation in a larger sample size including also severe cases is needed.

This study is the largest study to date investigating T-cell populations after SARS-CoV-2 infection and COVID-19 vaccination. Strengths include unbiased sampling as well as various SARS-CoV-2 infection characterization strategies, which allowed for the inclusion of both asymptomatic and symptomatic infected participants. The current study has a high percentage of participants infected during pregnancy (n=102, 29.1%), possibly due to sampling between April 2021 and October 2022 - a period during which NYC re-opened following quarantine (July 2021) - which could have led to an increase in SARS-CoV-2 infection rates compared to the year before when quarantine regulations were stricter. To account for various waves of the SARS-CoV-2 virus (beta, delta, omicron), with varying disease severity, health regulations and lifestyle consequences we controlled for time since the start of the COVID-19 pandemic (March 1, 2020). This study has several limitations. First, due to limited research activities during the pandemic, PBMC collection was initiated one year after initial sample collection as part of a study protocol modification, referred to as Generation C II. As a result, PBMCs were collected for a subset of the full cohort. The prevalence of SARS-CoV-2 infection and COVID-19 vaccination during pregnancy was higher in the current sample compared to excluded participants, possibly due to increased hospital visits as well as timing of the cohort during the second year of the pandemic when quarantine and masking regulations were less strict, and vaccines were widely available. Next, knowledge of the timing of infection for cases whose SARS-CoV-2 infection status was determined based on positive antibodies (n=75, 74%) was limited and estimated based on sampling date. This may have led to misclassification of SARS-CoV-2 infection timing. In addition, inherent to observational study design, the exposures were not controlled and the range between first evidence of SARS-CoV-2 infection and sampling was large (0 days to 2.5 years). Moreover, despite measures to minimize variance between batches (experiments performed by same researcher and according to protocol), additional factors that were not controlled for may have resulted in batch effects. Potential confounding factors include time between sample collection and sample processing, SARS-CoV-2 virus type and pre-existing auto-immune disease. Furthermore, we performed flow cytometry on cryopreserved cells and stained for cell-surface markers with no prior cell stimulation. Cell stimulation has been used in other recent COVID-19 studies in pregnancy, but is a relatively new method and needs further validation. Lastly, while the Generation C study is a multi-ethnic and socio-economically diverse urban pregnancy cohort, it is unique and may not be generalizable to other more ethnically/socio-economically homogeneous or rural populations.

The impact of SARS-CoV-2 infection on vulnerable populations, including pregnant people, has been a major concern since the COVID-19 outbreak. Early pandemic studies reported an increased risk of adverse pregnancy outcomes, such as preterm birth and stillbirth, following SARS-CoV-2 infection (30). More recent studies have suggested that initial findings were largely driven by severe illness and found no increased risk of preterm birth in asymptomatic or mild cases (31, 32). While we find no apparent T-cell changes following prenatal SARS-CoV-2 infection overall, considering the high inter-individual variance and the increased trend of the Th17/Treg ratio in repeated measurements following infection, as well as the impact of SARS-CoV-2 infection pre-pregnancy on the Th17/Treg ratio, we conclude that SARS-CoV-2 infection may impact these T-cell populations. Further validation studies including severely infected pregnant people, increased sample sizes including more participants per gestational age, and detailed information on pre-existing autoimmune disease are needed to draw final conclusions. Additionally, based on our finding that Th17 and Treg percentages vary between individuals but are rather consistent within an individual imply that future studies would benefit from longitudinal sampling to analyze trajectories, rather than a case-control analysis. Future research should aim to determine SARS-CoV-2 infection based on positive PCR testing to understand both acute and long-term effects, as well as investigate the acute T-cell response to SARS-CoV-2 infection, e.g. by restimulation of PBMCs after collection. Our finding of no apparent T-cell changes following prenatal COVID-19 vaccination is reassuring for the pregnant population and their caregivers.

## Data availability statement

The raw data supporting the conclusions of this article will be made available by the authors, without undue reservation.

## Ethics statement

The studies involving humans were approved by the Icahn School of Medicine at Mount Sinai Institutional Review Board (IRB-20-03352), reviewed by the US Centers for Disease Control and Prevention (CDC), and consistent with applicable federal law and CDC policy. The studies were conducted in accordance with the local legislation and institutional requirements. The participants provided their written informed consent to participate in this study.

## Author contributions

FG: Conceptualization, Formal analysis, Investigation, Validation, Visualization, Writing – original draft, Writing – review & editing. MG: Conceptualization, Formal analysis, Investigation, Methodology, Visualization, Writing – original draft, Writing – review & editing. JC: Writing – review & editing. CL: Investigation, Software, Writing – review & editing. A-SR: Conceptualization, Funding acquisition, Writing – review & editing. TF: Writing – review & editing. MP-R: Conceptualization, Funding acquisition, Writing – review & editing. SD: Funding acquisition, Writing – review & editing. JS: Funding acquisition, Writing – review & editing. TJ: Funding acquisition, Writing – review & editing. WL: Funding acquisition, Writing – review & editing. VB: Conceptualization, Funding acquisition, Supervision, Writing – review & editing. LD: Conceptualization, Supervision, Writing – original draft, Writing – review & editing.

## Supplementary material

Supplementary Figure 1 Study design. Flowchart of the sample selection from the Generation C cohort conducted in the Mount Sinai Health System in New York City, USA. 351 participants were included in the current analysis. Peripheral blood monocyte cells = PBMC.

Supplementary Figure 2 Flow cytometry gating strategy for identification of Th17 and Treg cells. **(A)**. PBMCs were isolated from the blood of pregnant participants and stained with monoclonal antibodies. Treg cells were gated based on CD3+CD4+CD25+CD127- expression. Th17 cells were determined based on the expression of CD3+CD4+CCR4+CCR6+CXCR3-CCR10-.

Supplementary Figure 3 Outlier analysis. Principal component analysis was performed to identify outlier samples. The line indicates a 95% confidence interval. Samples were excluded if they deviated more than three standard deviations (>3 SD) from the grand mean of the first principal component (PC1) (n=8). The remaining 455 samples were included in the current analysis.

Supplementary Figure 4 Comparison of outcomes based on time between SARS-CoV-2 infection/COVID-19 vaccination and sampling. **(A)** Percentage of CD4+, Th17, Treg and the Th17/Treg ratio compared between each infection timing group (0-3 months, 3-6 months, 6-9 months, 9-12 months, > 12 months between SARS-CoV-2 infection and sampling) and a reference group of never infected participants (REF). **(B)** Percentage of CD4+, Th17, Treg and the Th17/Treg ratio compared between each vaccine timing group (0-3 months, 3-6 months, 6-9 months, 9-12 months, > 12 months between COVID-19 vaccination and sampling) and a reference group of never vaccinated participants (REF).
