# Supplementary figures and images for: The effect of SARS-CoV-2 infection and vaccination on Th17 and regulatory T cells in a pregnancy cohort in NYC
Source: Front Immunol. 2024 Mar 5;15:1350288. doi: 10.3389/fimmu.2024.1350288 (PMC10948419; doi:10.3389/fimmu.2024.1350288)

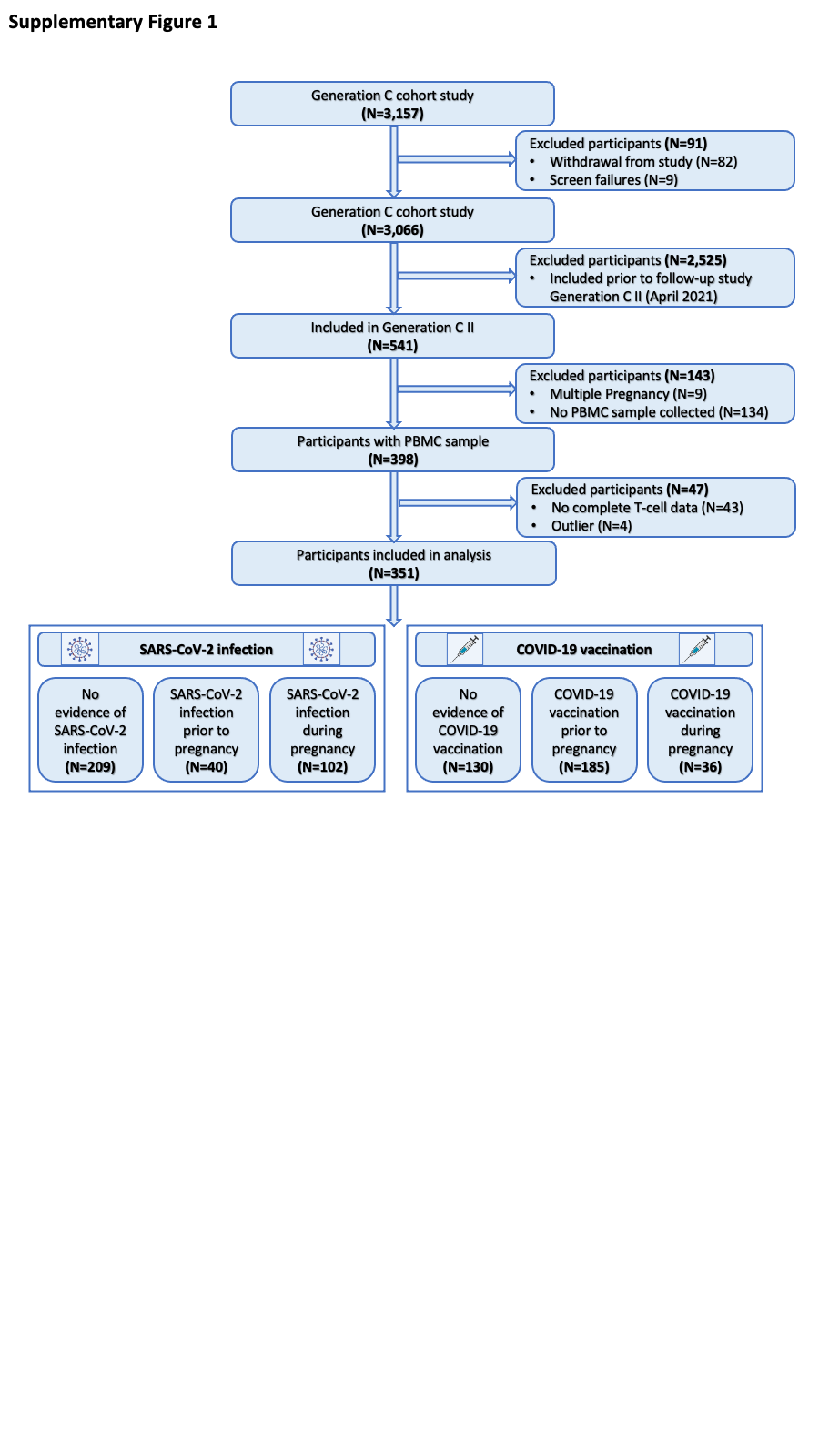

Supplement: Supplementary Figure 1 — Study design. Flowchart of the sample selection from the Generation C cohort conducted in the Mount Sinai Health System in New York City, USA. 351 participants were included in the current analysis. Peripheral blood monocyte cells = PBMC. [file Image_1.tiff]

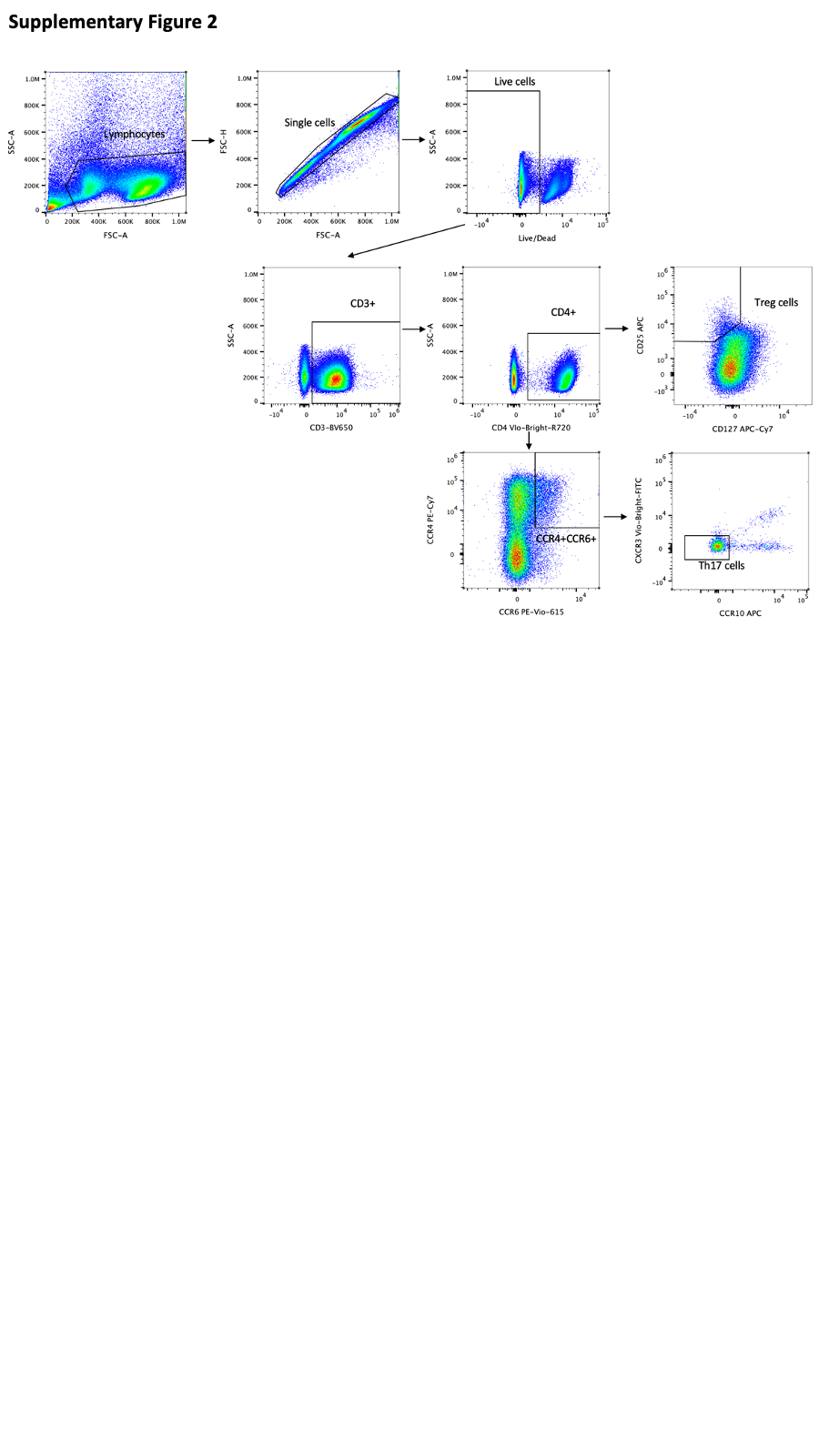

Supplement: Supplementary Figure 2 — Flow cytometry gating strategy for identification of Th17 and Treg cells. (A). PBMCs were isolated from the blood of pregnant participants and stained with monoclonal antibodies. Treg cells were gated based on CD3+CD4+CD25+CD127- expression. Th17 cells were determined based on the expression of CD3+CD4+CCR4+CCR6+CXCR3-CCR10-. [file Image_2.tiff]

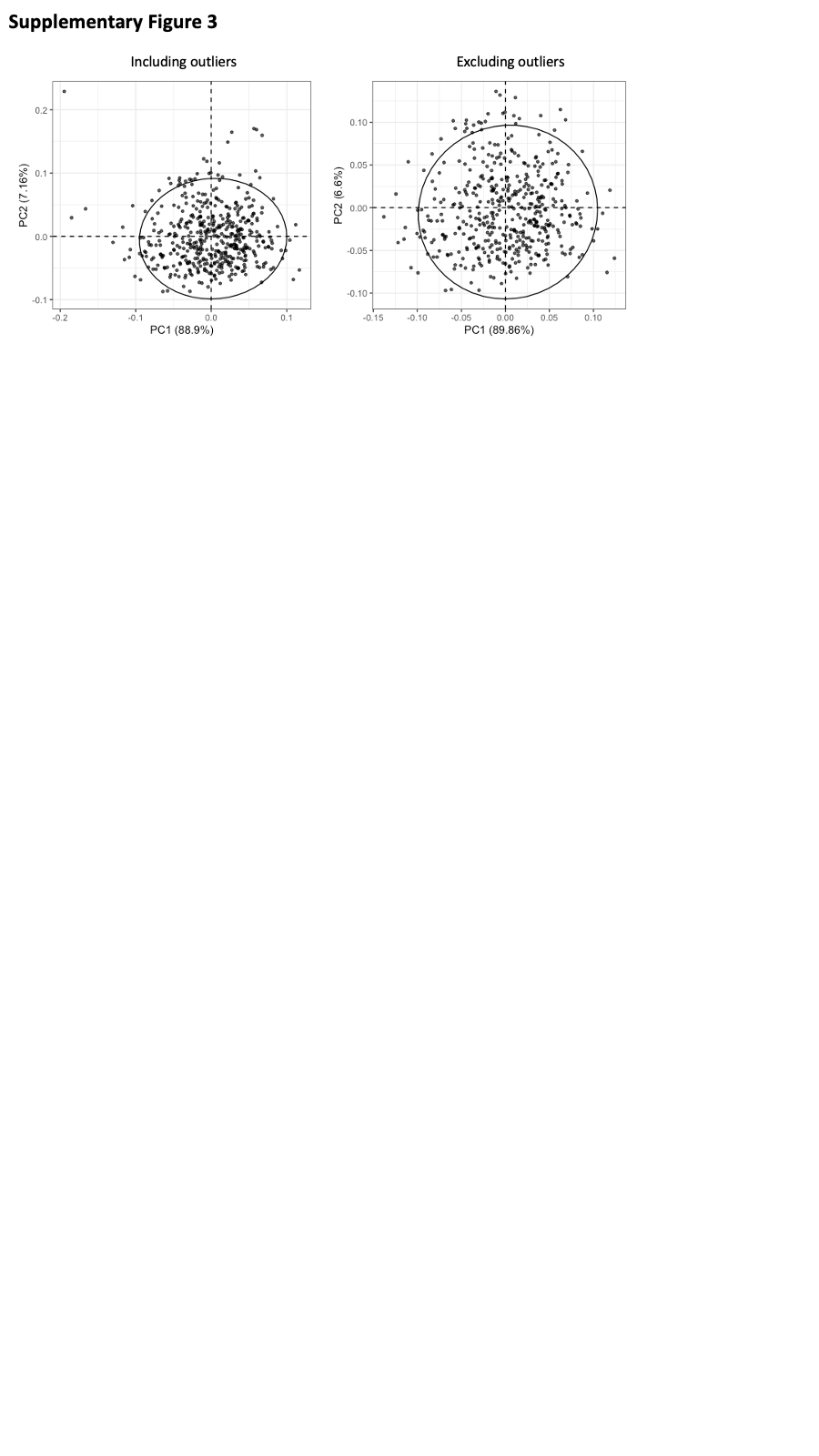

Supplement: Supplementary Figure 3 — Outlier analysis. Principal component analysis was performed to identify outlier samples. The line indicates a 95% confidence interval. Samples were excluded if they deviated more than three standard deviations (>3 SD) from the grand mean of the first principal component (PC1) (n=8). The remaining 455 samples were included in the current analysis. [file Image_3.tiff]

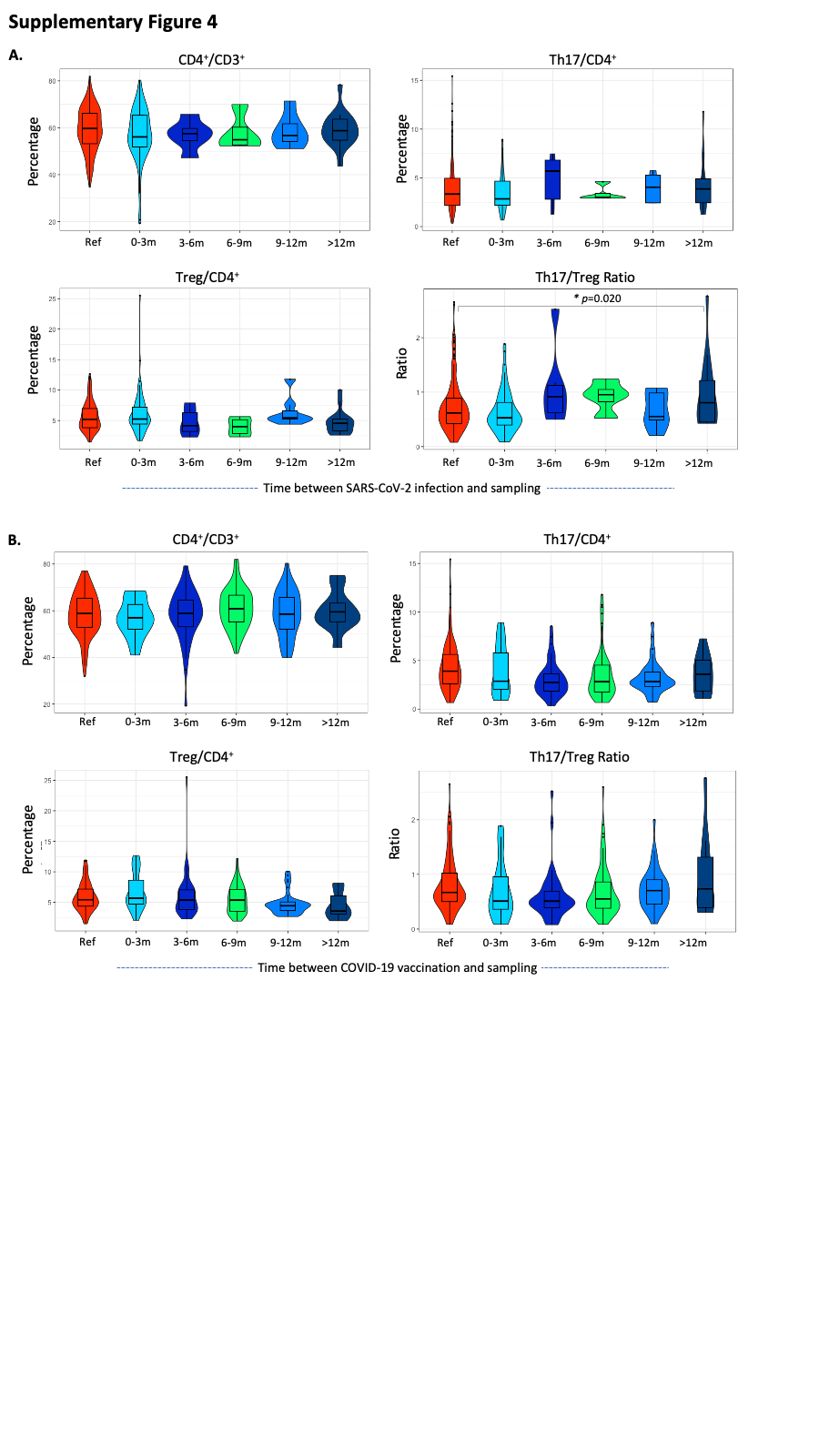

Supplement: Supplementary Figure 4 — Comparison of outcomes based on time between SARS-CoV-2 infection/COVID-19 vaccination and sampling. (A) Percentage of CD4+, Th17, Treg and the Th17/Treg ratio compared between each infection timing group (0-3 months, 3-6 months, 6-9 months, 9-12 months, > 12 months between SARS-CoV-2 infection and sampling) and a reference group of never infected participants (REF). (B) Percentage of CD4+, Th17, Treg and the Th17/Treg ratio compared between each vaccine timing group (0-3 months, 3-6 months, 6-9 months, 9-12 months, > 12 months between COVID-19 vaccination and sampling) and a reference group of never vaccinated participants (REF). [file Image_4.tiff]
