# Supplementary material for: The effect of SARS-CoV-2 infection and vaccination on Th17 and regulatory T cells in a pregnancy cohort in NYC
Source: Front Immunol. 2024 Mar 5;15:1350288. doi: 10.3389/fimmu.2024.1350288 (PMC10948419; doi:10.3389/fimmu.2024.1350288)
Supplement: Supplementary file 5 [file Table_1.docx]

**Supplementary Table 1** Comparison between the included sample (N=351) with participants included in the Generation C II follow up study (N=541) and the entire Generation C cohort (N=3,066).

| **Characteristic** | **PBMC sample (N=351)** | **Generation C II participants (N=541)** | **P-value** | **Generation C cohort (N=3,066)** | **P-value** |
| --- | --- | --- | --- | --- | --- |
| Maternal Age, median (IQR)   - <35 - 35 and up - Unknown | 211 (60.1)  97 (27.6)  43 (12.3) | 315 (58.2)  146 (27.0)  80 (14.8) | 0.053 | 1,853 (60.4)  918 (29.9)  295 (9.6) | 0.222 |
| Race/ethnicity, n (%)   - Asian - Black - Hispanic - White - Other - Unknown | 29 (8.3)  51 (14.5)  84 (23.9)  92 (26.2)  15 (4.3)  80 (22.8) | 48 (11.4)  70 (16.6)  122 (28.9)  150 (35.5)  26 (6.2)  125 (23.1) | 0.338 | 312 (10.8)  319 (14.6)  782 (27.2)  1,187 (51.9)  126 (4.4)  287 (9.4) | 0.099 |
| Pre-pregnancy BMI, n (%)   - Underweight (<18) - Normal weight (18-24.9) - Overweight (25-30) - Obese (>30) - Unknown | 11 (3.1)  153 (43.6)  78 (22.2)  100 (28.5)  9 (2.6) | 21 (3.9)  233 (43.1)  122 (22.6)  154 (28.5)  11 (2.0) | 0.952 | 85 (2.8)  1,325 (43.2)  843 (27.5)  762 (24.9)  51 (1.7) | 0.123 |
| Insurance, n (%)   - Private/Self-pay - Public | 248 (70.7)  103 (29.3) | 380 (70.2)  161 (29.8) | 0.671 | 786 (25.6) | 0.147 |
| SARS-CoV-2 infection status, n (%)   - Never infected - Infected prior to pregnancy - Infected during pregnancy | 209 (59.5)  40 (11.4)  102 (29.1) | 349 (64.5)  60 (11.1)  132 (24.4) | **0.004** | 2,398 (78.3)  93 (3.0)  570 (18.6) | **<0.001** |
| COVID-19 vaccination, n (%)   - No proof of vaccination prior to sampling - First dose prior to pregnancy - First dose during pregnancy prior to sampling | 130 (37.0)    185 (52.7)    36 (10.3) | 238 (44.0)  230 (42.5)  73 (13.5) | **<0.001** | 2,668 (87.5)  237 (7.8)  144 (4.7) | **<0.001** |

**Supplementary Table 2. T cell populations in all samples (N=455 samples) as well as across SARS-CoV-2 infection group.** The percentage of CD4^+^, Th17 and Treg cells, the Th17/Treg ratio and IL-17 levels were compared between samples collected from participants with no evidence of prior SARS-CoV-2 infection (N= 274 samples) and participants with SARS-CoV-2 infection prior to (N= 44 samples) or during pregnancy (N= 137 samples).

| **Characteristic** | **Overall (N= 455 samples)** | **Never infected (N=274)** | **SARS-CoV-2 infection prior to pregnancy (N=44)** | **SARS-CoV-2 infection during pregnancy (N=137)** | ***P*-value*** | ***P*-value**** |
| --- | --- | --- | --- | --- | --- | --- |
| Percentage CD4+ (CD4+/CD3+*100%), median (IQR) | 59.50 [53.00, 65.70] | 59.75 [52.92, 66.18] | 57.00 [53.88; 63.85] | 59.40 [52.70, 65.60] | 0.734 | 0.734 |
| Percentage Th17 (Th17/CD4+*100%), median (IQR) | 3.22 [2.12, 4.64] | 3.22 [2.11, 4.48] | 3.20 [2.42; 4.98] | 3.21 [2.07, 4.63] | 0.635 | 0.734 |
| Percentage Treg (Treg/CD4+*100%), median (IQR) | 5.04 [3.79, 6.69] | 5.08 [3.89, 6.96] | 4.25 [3.04; 5.39] | 5.07 [3.69, 6.54] | **0.009** | **0.045** |
| Ratio TH17/Treg (Th17/Treg*100%), median (IQR) | 0.60 [0.43, 0.89] | 0.6 [0.41, 0.85] | 0.85 [0.47; 1.10] | 0.57 [0.42, 0.84] | **0.041** | 0.103 |
| IL-17 (pg/ml), median (IQR) | 9.16 [6.83, 12.99] | 9.27 [6.77; 13.30] | 8.76 [5.78, 12.30] | 9.32 [7.00; 12.47] | 0.612 | 0.734 |

*P-value indicates statistical comparison between infection groups using Kruskal-Wallis test for non-normally distributed continuous variables.

**P-value after multiple testing correction

**Supplementary Table 3. T cell populations in all samples (N=455 samples) as well as across COVID-19 vaccination group.** The percentage of CD4+, Th17 and Treg cells and the Th17/Treg ratio were compared between samples collected from participants with no evidence of COVID-19 vaccination (N=166 samples), participants who received a first dose prior to pregnancy (N=246 samples) and participants who received the first dose during pregnancy (N=43 samples).

| **Characteristic** | **Overall (N=455 samples)** | **Never vaccinated**  **(N=166 samples)** | **First dose prior to pregnancy (N=246 samples)** | **First dose during pregnancy (N=43 samples)** | ***P*-value*** | ***P*-value**** |
| --- | --- | --- | --- | --- | --- | --- |
| Percentage CD4+, mean (SD) (CD4+/CD3*100%) | 59.50 [53.00, 65.70] | 58.35 [52.80, 64.45] | 60.55 [54.82, 66.20] | 55.90 [50.30, 65.50] | **0.010** | **0.02** |
| Percentage Th17, mean (SD) (TH17/CD4+*100%) | 3.22 [2.12, 4.64] | 3.71 [2.49, 5.32] | 2.84 [1.90, 4.07] | 3.65 [2.01, 4.97] | **<0.001** | **0.005** |
| Percentage Treg, mean (SD) (Treg/CD4+*100%) | 5.04 [3.79, 6.69] | 5.34 [4.19, 7.02] | 4.73 [3.60, 6.52] | 5.47 [4.53, 7.04] | **0.012** | **0.02** |
| Ratio TH17/Treg, mean (SD) (TH17/Treg*100%) | 0.60 [0.43, 0.89] | 0.66 [0.49, 0.97] | 0.57 [0.39, 0.83] | 0.6 [0.44, 0.88] | **0.025** | **0.031** |
| IL-17 (pg/ml), median (IQR) | 9.16 [6.83, 12.99] | 9.11 [6.55, 14.06] | 9.16 [6.84, 12.36] | 9.43 [7.59, 14.06] | 0.605 | 0.605 |

*P-value indicates statistical comparison between three vaccination groups using Kruskal-Wallis test for non-normally distributed continuous variables.

**P-value after multiple testing correction using the Benjamini Hochberg correction.

**Supplementary Table 4A. T cell populations based on time between SARS-CoV-2 infection and sampling.** SARS-CoV-2 infected participants were grouped according to the time between SARS-CoV-2 infection and sampling; 1) 0-3 months (N=61), 2) 3-6 months (N=7), 3) 6-9 months (N=4), 4) 9-12 months (N=8), 5) >12 months after SARS-CoV-2 infection (N=20). Participants were compared to a reference group of uninfected participants, or samples that were drawn prior to SARS-CoV-2 infection occurred (N=251). The percentage of CD4+, Th17 and Treg cells and the Th17/Treg ratio were compared between each group and the reference group.

| Time between SARS-CoV-2 infection and sampling | Percentage CD4+ (CD4+/CD3*100%) | | Percentage Th17 (TH17/CD4+*100%) | | Percentage Treg (Treg/CD4+*100%) | | Ratio TH17/Treg (TH17/Treg*100%) | |
| --- | --- | --- | --- | --- | --- | --- | --- | --- |
|  | Estimate | P-value* | Estimate | P-value* | Estimate | P-value* | Estimate | P-value* |
| 0-3 months^a^ | -4.4 (2.3) | 0.310 | -0.2 (0.5) | 0.848 | 0.8 (0.6) | 0.468 | -0.1 (0.1) | 0.468 |
| 3-6 months^a^ | -3.9 (7.4) | 0.848 | 1.1 (1.6) | 0.848 | -1.2 (1.8) | 0.848 | 0.7 (0.3) | 0.100 |
| 6-9 months^a^ | -2.5 (10.2) | 0.848 | -0.7 (2.2) | 0.848 | -4.0 (2.5) | 0.468 | 0.1 (0.4) | 0.848 |
| 9-12 months^a^ | -10.8 (10.3) | 0.651 | 0.8 (2.2) | 0.848 | -3.6 (2.6) | 0.468 | 0.3 (0.4) | 0.848 |
| >12 months^a^ | -0.7 (4.6) | 0.874 | 0.3 (0.9) | 0.848 | -2.2 (1.2) | 0.310 | 0.6 (0.2) | **0.020** |

^a^ Each group was compared to a reference group of participants with no evidence of SARS-CoV-2 infection.

^*^Analyses were adjusted for maternal age (<35, >35), BMI (underweight (<18), normal weight (18-25), overweight (25-30), obese (>30)), race/ethnicity (Black, Asian, Hispanic, White, Other), parity (nulliparous/multiparous), insurance (private/self-pay, public), pre-pregnancy diabetes (yes/no), pre-pregnancy hypertension (yes/no), fetal sex (male/female), time since the start of the pandemic (weeks since first day of pregnancy and March 1, 2020), time between first evidence of SARS-CoV-2 infection and sampling, COVID-19 vaccination status (no evidence of prior vaccination, first dose prior to pregnancy, first dose during pregnancy) and technical covariates gestational age at sampling (days), experiment batch and compensation batch. Analyses were corrected for multiple testing using Benjamini Hochberg.

**Supplementary Table 4B. T cell populations based on time between COVID-19 vaccination and sampling.** COVID-19 vaccinated participants were grouped according to the time between COVID-19 vaccination and sampling; 1) 0-3 months (N=22), 2) 3-6 months (N=49), 3) 6-9 months (N=74), 4) 9-12 months (N=46), 5) >12 months after SARS-CoV-2 infection (N=11). Participants were compared to a reference group of samples obtained from unvaccinated participants, or samples that were drawn prior to COVID-19 vaccination occurred (N=149).The percentage of CD4+, Th17 and Treg cells and the Th17/Treg ratio were compared between each group and the reference group.

|  | Percentage CD4+ (CD4+/CD3*100%) | | Percentage Th17 (TH17/CD4+*100%) | | Percentage Treg (Treg/CD4+*100%) | | Ratio TH17/Treg (TH17/Treg*100%) | |
| --- | --- | --- | --- | --- | --- | --- | --- | --- |
| Time between COVID-19 vaccination and sampling | Estimate | P-value* | Estimate | P-value* | Estimate | P-value* | Estimate | P-value* |
| 0-3 months^a^ | -2.3 (4.2) | 0.824 | -0.1 (0.9) | 0.993 | -1.2 (1.0) | 0.595 | 0.1 (0.2) | 0.792 |
| 3-6 months^a^ | -3.9 (2.4) | 0.500 | -0.8 (0.5) | 0.500 | 1.2 (0.6) | 0.500 | -0.2 (0.1) | 0.500 |
| 6-9 months^a^ | 2.0 (2.9) | 0.792 | 0.1 (0.1) | 0.993 | 0.1 (0.7) | 0.993 | -0.1 (0.1) | 0.989 |
| 9-12 months^a^ | -1.7 (2.6) | 0.798 | -1.1 (0.6) | 0.500 | -0.9 (0.7) | 0.583 | -0.1 (0.1) | 0.792 |
| >12 months^a^ | 0.9 (3.6) | 0.987 | -1.0 (0.8) | 0.583 | -0.8 (0.9) | 0.769 | 0.1 (0.2) | 0.993 |

^a^ Each group was compared to a reference group of participants with no COVID-19 vaccination.

^*^Analyses were adjusted for maternal age (<35, >35), BMI (underweight (<18), normal weight (18-25), overweight (25-30), obese (>30)), race/ethnicity (Black, Asian, Hispanic, White, Other), parity (nulliparous/multiparous), insurance (private/self- pay, public), pre-pregnancy diabetes (yes/no), pre-pregnancy hypertension (yes/no), fetal sex (male/female), time since the start of the pandemic (weeks since first day of pregnancy and March 1, 2020), time between first evidence of COVID-19 vaccination and sampling, SARS-CoV-2 infection status (no evidence of prior infection, infected prior to pregnancy, infected during pregnancy) and technical covariates gestational age at sampling (days), experiment batch and compensation batch. Analyses were corrected for multiple testing using Benjamini Hochberg.
